# Late Presentation of a Childhood Neuroblastoma in an Adult Patient

**DOI:** 10.7759/cureus.72438

**Published:** 2024-10-26

**Authors:** Intisar Al Hashimi, Khurram Siddiqui, Ghalib Al Badaai, Suad Al Jahdhami

**Affiliations:** 1 Urology, Sultan Qaboos University Hospital, Muscat, OMN; 2 Pathology, Sultan Qaboos University Hospital, Muscat, OMN

**Keywords:** incidence, ivc involvement, mycn amplification, neuroblastome, staging

## Abstract

This case report details a rare presentation of neuroblastoma in an adult patient. The patient, a 22-year-old female, presented with a right-sided abdominal mass and discomfort for the last 12 months. Imaging studies revealed a huge adrenal mass occupying the right quadrant of the abdomen with displacement of the surrounding structures. Intraoperatively, the mass was adherent to the liver and very close to the inferior vena cava. The mass was resected completely without any intraoperative complications. The histopathological examination of the mass revealed a poorly differentiated neuroblastoma. This report highlights a rare presentation of neuroblastoma in the adult population.

## Introduction

Although neuroblastoma is the most common extra-cranial solid tumor in childhood, it is rare in adults [1-3]. It is derived from the neural crest cells of the sympathetic nervous system and typically develops in the adrenal medulla or paraspinal sympathetic ganglia. Neuroblastoma usually occurs sporadically, but familial cases are also observed [4,5]. It has a remarkable phenotypic heterogeneity that can show many different chromosomal abnormalities. Neuroblastomas most commonly develop in the abdomen and are most frequently localized in the adrenal gland. Neuroblastoma has different clinical courses, from metastatic spread to spontaneous regression [4,6]. Advanced disease at presentation is common. Diagnosis of neuroblastoma is made based on histopathological examination. This report discusses a unique case of neuroblastoma diagnosed at the age of 22 years with a huge mass occupying the entire right quadrant of the abdomen.

## Case presentation

A 22-year-old female known to have beta-thalassemia trait presented with lethargy, anemia, and a right-sided abdominal mass associated with bloating and abdominal pain. The pain started one year ago, and it was dull in nature. It was localized to the right upper quadrant of the abdomen. There was an undocumented history of adrenal mass diagnosed in childhood which was not followed up. She reported that this mass was increasing in size gradually. She did not report any headaches, palpitations, or high blood pressure. There was no history of malignancy in the family. Physical examination revealed a thin-built lady. She was pale and tachycardic. The abdominal examination revealed a very large abdominal mass occupying the right upper quadrant and extending to the right lower quadrant. It was hard in consistency, non-tender, and there were no overlying skin changes.

Blood investigations are shown in Table 1.

**Table 1:** Laboratory investigations results

Investigations	Result	Reference Range
Hemoglobin	6.9 g/dL	11.0-14.5 g/dL
White Cell Count	5 × 10^9^/L	2.4-9.5 × 10^9^/L
C-Reactive Protein	122 mg/L	0-5 mg/L

**Figure 1:**
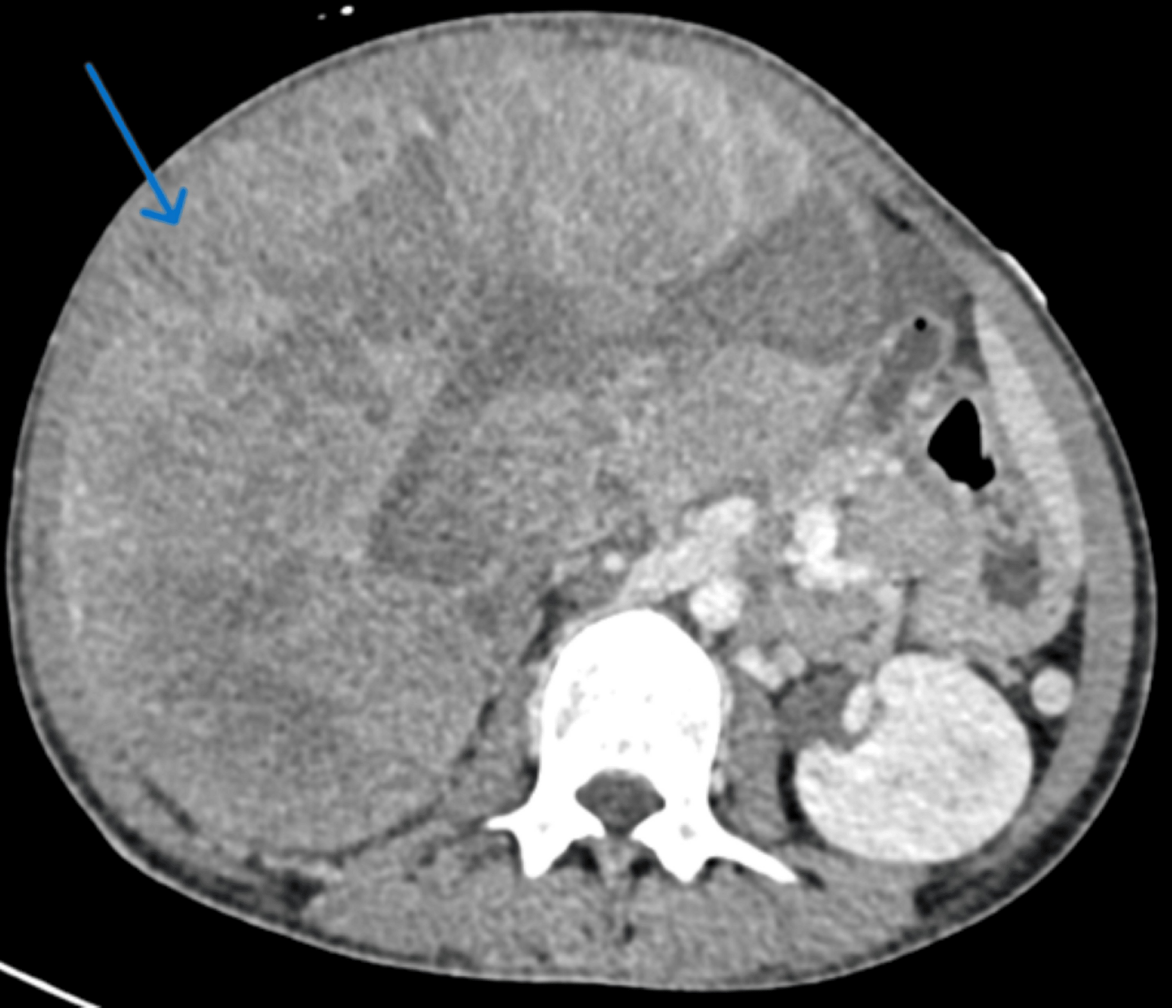
CT scan of the abdomen showing a very large heterogeneous mass

**Figure 2:**
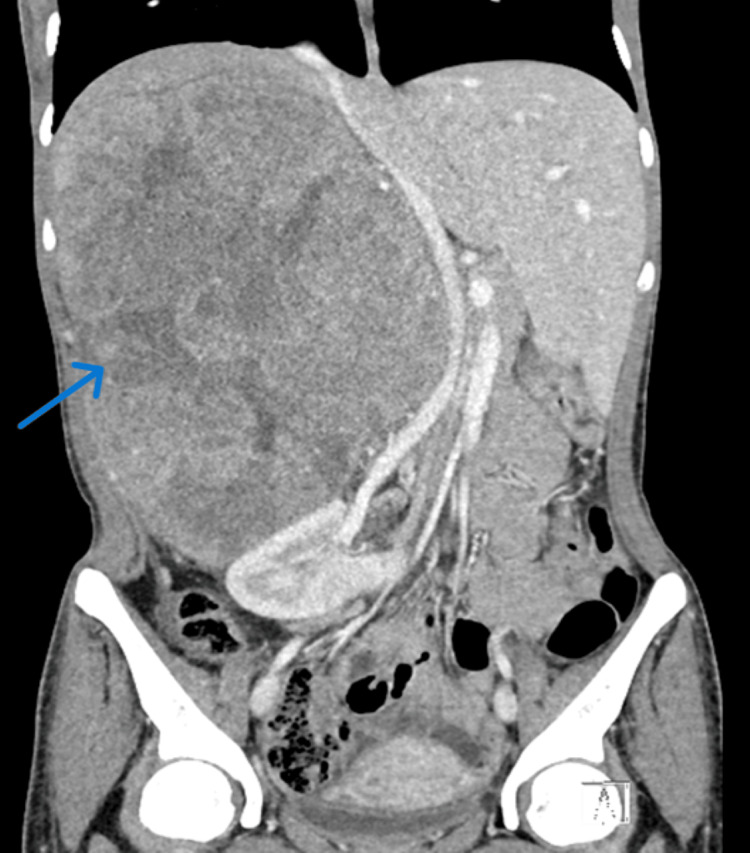
Coronal view of the CT scan of the abdomen showing the proximity of the mass to the IVC and displacement of the IVC, liver, and right kidney IVC: inferior vena cava

A contrast-enhanced computed tomography of the abdomen and pelvis demonstrated a right suprarenal heterogeneous solid mass measuring 24 × 19 × 17 cm in diameter (Figure 1). The mass had a heterogeneous enhancement with central necrosis. There were two foci of macroscopic calcifications and a mass effect on the all-adjacent structures. The mass was displacing the inferior vena cava without any signs of invasion (Figure 2). Subsequently, the 24-hour urine catecholamine excretion was done and was within normal limits. 

A fluorodeoxyglucose-positron emission tomography (FDG PET) scan was done which showed a right-sided abdominal mass of an adrenal origin, likely to be a neuroendocrine mass. There was no evidence of lymph node involvement. An incidental finding of an enlarged thyroid with increased diffused uptake was noted. Thyroid-stimulating hormone (TSH) level was checked and the value was 6.00 mIU/L (0.27 - 4.20).

**Figure 3:**
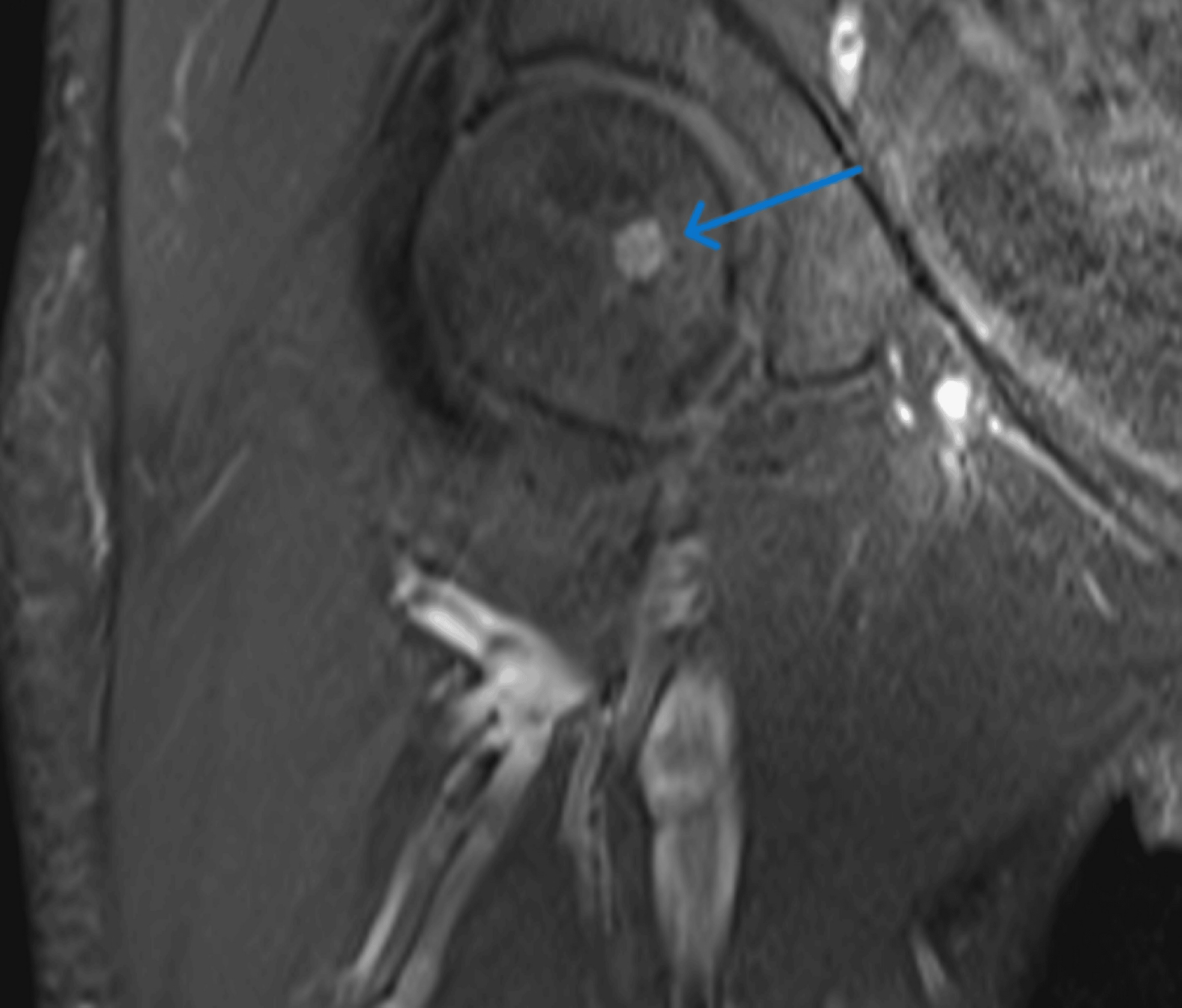
MRI of the hip showing a 5-mm enhancing lesion at the right femoral head

A Gallium-68 DOTATATE scan was performed and was suspicious for pheochromocytoma with mildly increased uptake in the left head of the femur. An MRI of the hip was also done which showed a solitary 5 mm enhancing well-defined lesion at the right femoral head, this lesion could represent an occult metastatic disease (Figure 3).

After optimization of the patient’s condition, a laparotomy was carried out. Intraoperatively, the mass was adherent to the liver, it was carefully dissected away. The mass was separated from the surrounding structures including the kidney and the inferior vena cava. There were no intraoperative complications.

**Figure 4:**
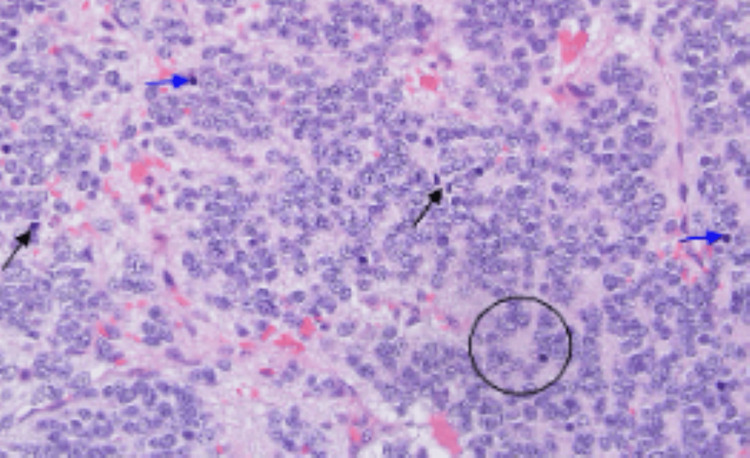
Hematoxylin & eosin-stained section of adrenal gland tumor revealing features of poorly differentiated neuroblastoma Mitotic figures (black arrows), apoptotic nuclei (blue arrows), and focal Homer-Wright rosette were also noted (circle) (×400).

**Figure 5:**
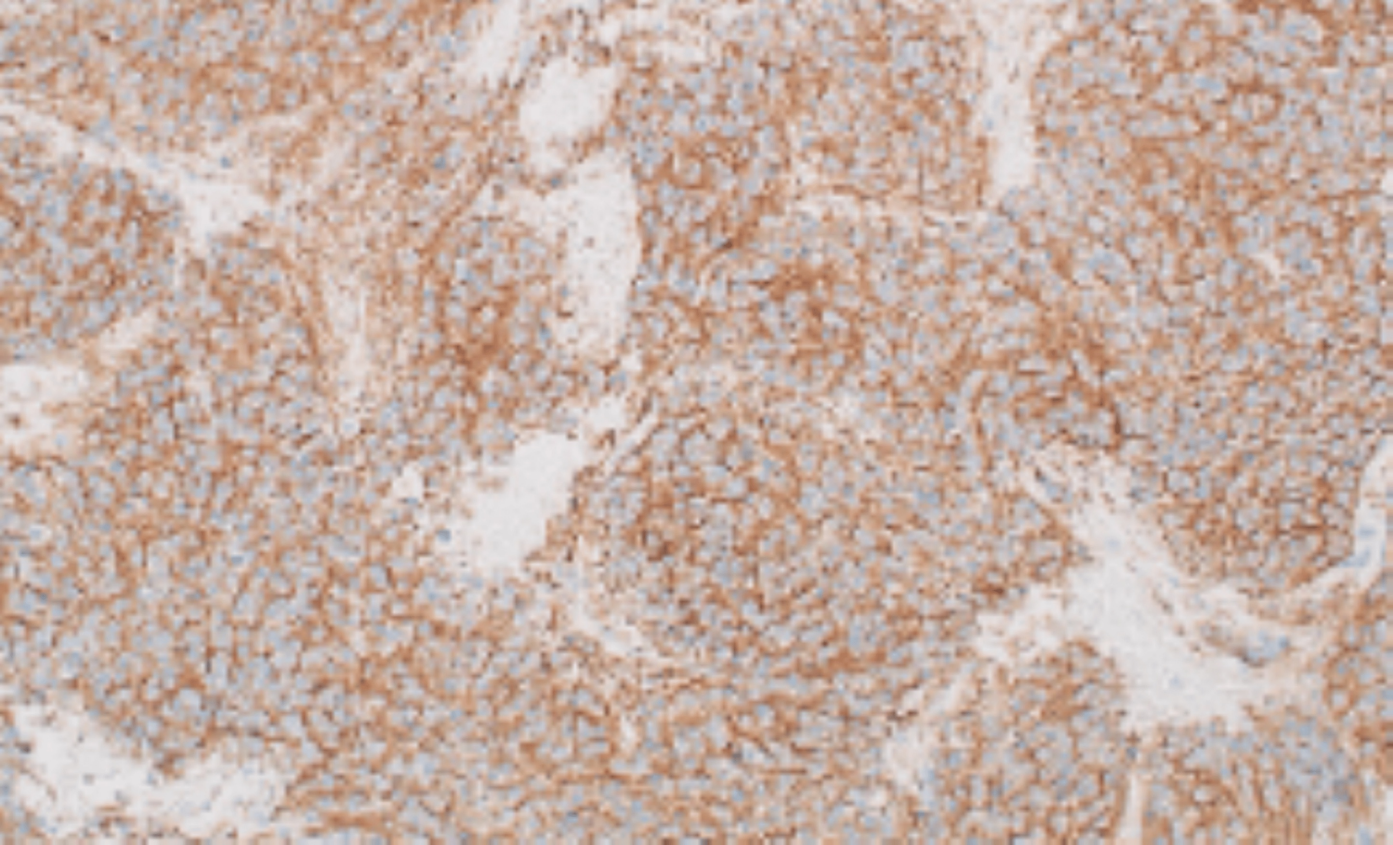
Synaptophysin immunohistochemical stain is diffusely positive in tumor cells

The histopathological examination of the mass revealed a poorly differentiated neuroblastoma (Figures 4-5). There was evidence of lymph vascular space invasion. The surgical margin was negative and there was no lymph node involvement. It was negative for MYCN amplification. Postoperatively and after a discussion of the case in the multidisciplinary team meeting, the patient was referred to the oncology center for ^123^I-metaiodobenzylguanidine (MIBG) scintigraphy and possible chemotherapy. Unfortunately, the patient refused further treatment. On telephonic follow-up at 1 year, the patient seems to be doing well with no complaints.

## Discussion

Neuroblastoma is a rare tumor. It represents approximately 7% of all cases of childhood cancer and results in about 15% of cancer deaths in children [7]. It was first described by Dr. Rudolf Virchow as a “glioma” in the abdominal cavity [8]. It has been estimated that more than 700 patients are diagnosed with neuroblastoma annually in the United States [9]. The average age of a patient at the time of a neuroblastoma diagnosis is between one and two years of age. Less than 5% of neuroblastomas are diagnosed at 10 years of age or older [10]. This patient was diagnosed at the age of 22. Neuroblastoma in the adult population is a rare finding, with a worse prognosis compared to pediatric patients [11]. The average incidence of adult neuroblastoma is predicted to be one per 10 million adults per year [12]. A study was done in the United States to compare the incidence of neuroblastoma in children and adults showed that 6.1% of neuroblastomas were seen in patients more than 20 years of age and only 0.9% of neuroblastomas were diagnosed in patients more than 60 years of age [13]. Mosse et al. [14] found that the older the age at the time of diagnosis, the worse the prognosis and the overall survival. It was also related to unfavorable staging scores.

Neuroblastoma may be sporadic or nonfamilial in origin [4]. However, familial cases have been also identified. Although its etiology is not well understood, the recent genome and family-based studies have improved the understanding of the genetic susceptibility to neuroblastoma [15].

In this patient, the mass was displacing the inferior vena cava without invasion. A review of the literature showed very few cases describing abdominal neuroblastomas with extension into the inferior vena cava [16-18]. Neuroblastoma is considered a complex and heterogeneous disease due to the diversity of presentation. Some tumors regress spontaneously while others are aggressive and metastatic and require multimodal treatment. Thus, signs and symptoms range from an asymptomatic palpable mass to a significant critical illness.

Staging is made based on the International Neuroblastoma Staging System. Neuroblastomas metastasize to the bone, bone marrow, lymph nodes, lungs, and liver. Hypertension is rare and is often caused by compression of the renal artery rather than the catecholamine excess. Patients’ risks are classified as low, intermediate, or high, based on age at diagnosis, stage defined by the extent of the disease by the INSS, MYCN proto-oncogene status, and DNA index [19,20].

This case indicates the importance of considering neuroblastoma as a differential diagnosis of adrenal masses in the adult population.

## Conclusions

Neuroblastoma is a tumor rarely seen in adults. This case report highlights the importance of considering neuroblastomas as a possible differential diagnosis of adrenal lesions in adults. This patient was successfully managed surgically without any complications. Further case reports are required to describe the presentation and the prognosis of neuroblastoma in adults.
